# Materials Engineering for Flexible Metallic Thin Film Applications

**DOI:** 10.3390/ma15030926

**Published:** 2022-01-25

**Authors:** Megan J. Cordill, Patrice Kreiml, Christian Mitterer

**Affiliations:** 1 Erich Schmid Institute for Materials Science, Austrian Academy of Sciences, 8700 Leoben, Austria; 2 Department of Materials Science, Montanuniversität Leoben, 8700 Leoben, Austria; christian.mitterer@unileoben.ac.at

**Keywords:** thin films, sputtering, electro-mechanical, flexible

## Abstract

More and more flexible, bendable, and stretchable sensors and displays are becoming a reality. While complex engineering and fabrication methods exist to manufacture flexible thin film systems, materials engineering through advanced metallic thin film deposition methods can also be utilized to create robust and long-lasting flexible devices. In this review, materials engineering concepts as well as electro-mechanical testing aspects will be discussed for metallic films. Through the use of residual stress, film thickness, or microstructure tailoring, all controlled by the film deposition parameters, long-lasting flexible film systems in terms of increased fracture or deformation strains, electrical or mechanical reliability, can be generated. These topics, as well as concrete examples, will be discussed. One objective of this work is to provide a toolbox with sustainable and scalable methods to create robust metal thin films for flexible, bendable, and stretchable applications.

## 1. Introduction

Thin films on rigid substrates are present in the applications and devices people use every day. The mechanical, thermal, electrical, and magnetic properties of thin films often come from the small dimensions. These small dimensional properties translate directly to flexible, stretchable, and bendable devices. From highly advanced foldable mobile phones and thermally resistant multilayers for aerospace applications to more simple uses in food packaging, thin films on compliant polymer substrates are ubiquitous. Some challenges with flexible and bendable applications include depositing films onto compliant and thinner polymer foils and how to properly characterize the electro-mechanical reliability of the flexible systems. Over the last two decades, new knowledge has been obtained about how different thin film deposition methods can be effectively used to fabricate metal and ceramic thin films on polymer substrates as well as advances in how to determine the mechanical and electrical limits of various flexible thin film systems. These methods are more sustainable, requiring no changes to existing thin film deposition infrastructure, as well as being more scalable compared to new, more complex manufacturing methods.

Reviews and other contributions are available about designing flexible thin film devices that focus on how the geometry of lines or thin film transistor (TFT) islands can be designed to create robust devices [1,2,3,4,5]. These advances include the use of meanders, nanowires or nanomeshes, conductive polymers, and wrinkling [6,7,8]. Here, the focus is to illustrate how metallic thin film engineering can be applied to create long-lasting, and even unbreakable, flexible systems. First, deposition methods to fabricate thin metal films for flexible electronic applications will be introduced, and next the challenges of growing these films on polymer substrates are highlighted (Section 2). Deposition methods are followed by a brief introduction into electro-mechanical testing methods for thin films on polymer substrates (Section 3). In Section 4, several thin metal film materials design examples are summarized, including film thickness, residual stress, microstructure tailoring, the significance of thin film architecture, and thin film alloying possibilities. Through these deposition-based strategies, reliable flexible metal films can be created without the need for complicated geometries or additional fabrication steps.

## 2. Thin Film Deposition

Nowadays, numerous methods are available for the deposition of functional thin films, being used for a plethora of applications, such as for wear protection of tools and components, as interconnect lines, adhesion or seed layers, semiconductor films, diffusion barriers, insulating layers and heat sinks in microelectronics, electrode layers with tunable optical and electrical properties in optoelectronics and displays, for energy harvesting in photovoltaics, as self-cleaning photocatalytic films for optical lenses or mirrors, or as permeation barriers for packaging foils. Many of these applications use physical vapor deposition (PVD) techniques, representing a versatile family of techniques for the deposition of high-quality films with tailored multifunctional properties. In particular, sputter deposition methods also enable the growth of thin films at temperatures close to room temperature, making them highly suitable for metallic film deposition on flexible polymer substrates. Today, the majority of thin films are deposited on metal, ceramic, semiconductor, and glass substrates, whereas recently, polymers have gained importance, due to their light weight (being advantageous for automotive applications) and their flexibility (making them highly suitable for wearable/flexible electronics). This section gives an overview on sputter deposition methods for growing thin metal films at low temperatures on flexible substrates, summarizing the present state-of-the-art methods and highlighting some of the challenges of the large-area coating of polymers. 

### 2.1. Plasmas in Thin Film Deposition

In PVD processes, the plasma plays a crucial role in enabling the growth of dense thin films at or at least close to room temperature. The plasma fulfills a two-fold task, (*i*) to generate a vapor phase, e.g., in sputtering, and (*ii*) to activate thin film growth processes occurring at the substrate. A plasma is generally a quasi-neutral gas containing ions, electrons, and neutrals, which shows a collective behavior in the presence of electromagnetic fields [9]. Technically, a plasma is generated by an electrical discharge, where a voltage is applied to two electrodes placed within a vacuum chamber, which is backfilled with a suitable process gas. As a result of the electrical field, positive and negative charge carriers drift in opposite directions and collisions may occur with all the particles present in the plasma. During *elastic* collisions, energy is transferred from one particle to another, with a change in the internal energy. *Inelastic* collisions between an electron of sufficient kinetic energy and a neutral may lead to ionization, leaving behind a positively charged ion and another electron. The average distance a particle can travel without a collision is called the *mean free path*, which is determined by the number of particles available (which is proportional to the pressure) and the cross-section of the respective collision. At the typical pressures of glow-discharges used for sputter deposition, namely 0.1–1 Pa, the mean free path is in the range of a few cm, with only a few collisions between energetic particles on their way from the vapor source (the cathode) to the substrate (the anode) with typical distances in the 10 cm range, and the process gas (commonly Ar) will occur.

The *degree of ionization*, specifically the fraction of ionized species within the plasma, is another important characteristic parameter. A cold plasma, such as is generated in a direct current (d.c.) glow-discharge used for sputter deposition, consists mostly of neutrals, with a degree of ionization, i.e., the number of ions divided by the numbers of ions and neutrals within the plasma, of only 10^−4^. In contrast, in a thermal plasma the current within the glow-discharge is increased until the voltage drops. This leads to the local confinement of the plasma within an arc discharge, as used for cathodic arc evaporation with its much higher degree of ionization.

Besides the fluxes of charge carriers within the discharge, the potential distribution plays a decisive role in determining plasma properties. A technical plasma, and therefore, the charge carriers present within the plasma, are in contact with grounded objects (e.g., the vacuum chamber) confining the plasma. There, as a result of the mass difference, the flux densities of electrons and ions arriving at surfaces in contact to the plasma are not equal, leading to the development of a potential difference repelling the faster electrons and attracting the slower ions towards the grounded object. Consequently, the plasma is characterized by a positive potential: the *plasma potential*. If the object in contact with the plasma is not grounded, the arriving electron current results in a negative charging, the *floating potential*, until a zero-net flux of ions and electrons is achieved. The floating potential is, for example, formed on an electrically insulating substrate surface; it is thus of vital importance to understand film growth on a non-conductive polymer surface.

### 2.2. Sputter Deposition

Sputtering is based on momentum transfer from positively charged highly energetic ions, in most cases Ar ions, to a solid target forming the vapor source (i.e., the cathode in the gas discharge) [10]. The kinetic energy of these Ar ions is given by their electrical charge (in sputtering, they are mainly single charged) and the cathode voltage applied, leading to values in the 100–1000 eV range. These ions impinging on the target surface result in a collision cascade, leading to the ejection of target surface atoms and creating a vapor phase. The low deposition rates of conventional diode sputtering and the excessive substrate heating by electron bombardment can be overcome by the use of magnetrons. There, arranging permanent magnets behind the target, the electrons, and thus the plasma, are confined by the superposition of the electrical field originating from the discharge and the magnetic field in front of the target. In a balanced magnetron, the magnetic field lines loop between the central and the outer magnets, providing an efficient plasma confinement. Unbalancing the magnetic field by providing outer magnets, which are stronger than the central ones, enables the plasma to expand away from the target area and a more pronounced interaction of energetic plasma species with the surface of the growing film [11].

The major benefit of sputtering is that virtually any solid material can be used as a target [12]. The thin film material spectrum can be even more expanded by reactive sputtering of a metallic target in an atmosphere containing reactive gases, such as N_2_ or O_2_, which leads to the deposition of nitride or oxide films, respectively. In such cases, a compound surface layer may also be formed on the target surface; this process is called target poisoning. This may lead to a significant reduction of the sputter yield, i.e., the number of target atoms sputtered per impinging ion, which is typically much lower for compounds than for metallic surfaces. Target poisoning can be overcome by the efficient control of the electrical power applied to the targets and the partial pressure of the reactive gas [13]. In many cases, sputtering is done using d.c. discharges, because of low-cost power supplies [14]. There, the formation of electrically less or non-conductive reaction layers, such as oxides, can lead to charging of the target surface and arcing. For electrically less conductive or insulating target materials, pulsed d.c. (operated in asymmetric bipolar mode in the few 10 to few 100 kHz range where short positive pulses result in de-charging of the target surface [15]) and radio-frequency power supplies are used.

### 2.3. Thin Film Growth

**Figure 1:**
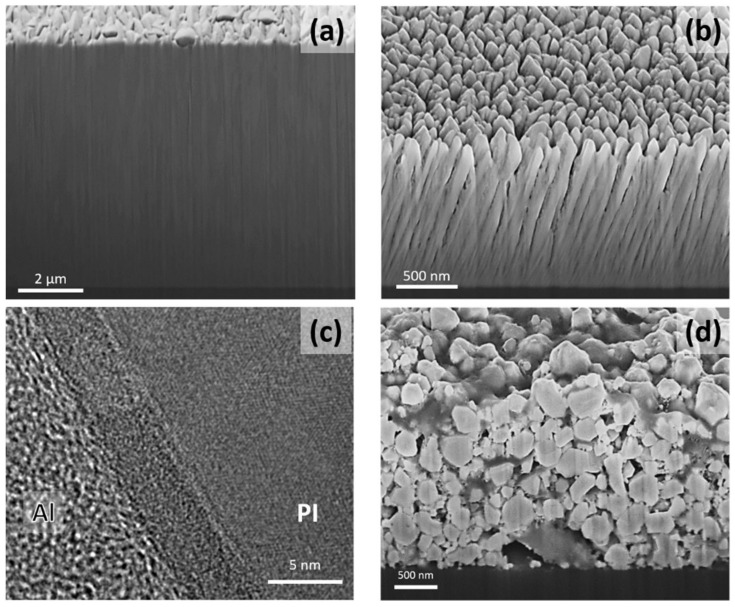
Cross-sections of thin films. (**a**) SEM micrograph illustrating the fine-columnar growth with columns perpendicular to the substrate surface of a film with a composition of Mo_0.72_Al_0.17_Ti_0.11_ deposited directly in front of a rotatable Mo_0.70_Al_0.20_Ti_0.10_ target and (**b**) the open-voided columnar growth of a film grown from the same target and using the same deposition parameters under inclined conditions resulting in a composition of Mo_0.67_Al_0.23_Ti_0.10_ [19]. (**c**) Transmission electron microscopy cross-section of an Al film thermally evaporated on a polyimide (PI) substrate, clearly indicating the modified near surface zone formed during film deposition (courtesy of B. Putz, C. Gammer, B. Völker). (**d**) SEM micrograph of a Cu film deposited by inkjet printing.

Film growth during sputter deposition is a highly non-equilibrium process, with thermodynamic restrictions stemming from low-temperature growth and kinetic restrictions due to high growth rates [16]. Energetic species arriving at the film surface condense at extremely high cooling rates of up to 10^13^ K/s [17], which leaves very little time for surface diffusion and finding preferential nucleation or growth sites, before the condensed atoms are overgrown by others. The growth of films with a metallic or ceramic character on polymer substrates typically follows the island growth mode (Volmer–Weber growth) [18], where stable clusters formed on the substrate surface grow in three dimensions. Ultimately, the coalescence of these islands occurs when neighboring islands get in contact, followed by the competitive growth of the grains. This growth mode leads to the formation of V-shaped columnar grains, with column boundaries typically crossing the complete film, as exemplified by the scanning electron microscopy (SEM) cross-section prepared by focused ion beam (FIB) milling in Figure 1a [19]. Island growth necessitates—depending on the combination of substrate and thin film material as well as the growth conditions—a certain thickness, before continuous films completely covering the whole substrate surface are formed. 

Since the early stages of PVD [20], thin film microstructures have been classified in structure zone models [21], where the thermal and kinetic activation leads to growth governed by the competition of atomic shadowing and diffusion processes. At low substrate temperatures, the less pronounced surface diffusion of the film forming species can be enhanced by atomic scale heating due to the kinetic energy of the species arriving at the film surface. This is typically promoted by low gas pressures, resulting in a high mean free path and less pronounced energy loss of sputtered species and by applying a negative bias voltage to the substrate, this accelerates the Ar^+^ ions to bombard the film surface [22].

### 2.4. Large-Area Deposition

A key for the success of sputter deposition is its scalability, enabling lab-scale to high-throughput large-area film deposition. Planar magnetrons have been dominating the last five decades, having either a round or rectangular shape, with target surface areas in the low cm^2^ range up to several m^2^. There, the plasma confinement in front of the target results in inhomogeneous target erosion, where only 20–40% of the target material are vaporized before the erosion groove approaches the target thickness [12,23]. For better target utilization, and—equally important—to reduce downtimes for target replacement in large-area deposition, sophisticated magnet configurations with magnetic fields varying over time are used [24]. In large-area deposition, rotatable cylindrical magnetrons, where a tube target is permanently rotating through the magnetic field, thus continuously sputtering a fresh target surface, are gaining importance. This enables on the one hand target utilization up to 80%, and, on the other hand, efficient target cooling allowing the application of high sputtering powers without overheating the target [25].

As a result of low process gas pressures, sputter deposition is a line-of-sight process, which means that only those areas facing the sputtered target surface will be coated. Using planar targets with their inherently inhomogeneous erosion leads to local thickness differences even in front of the target surface [23]. For both planar and rotatable magnetrons, the coating of large-area substrates (for example, the most recent 10.5 generation display glass dimension is 2940 × 3370 mm^2^ [26]) necessitates their permanent oscillation in front of the targets. This, however, results in periodic changes of the direction of the flux of film-forming species arriving at the substrate surface varying permanently between perpendicular and glancing angle deposition, which will also cause periodic variations of the growth rate. In addition, the kinetic energy of these species varies cyclically with the permanent variation of the distance between target and substrate, which affects the number of collisions of sputtered species with the process gas. Consequently, film growth on oscillating substrates varies from a flux of highly energetic species arriving perpendicular to the substrate surfaces to a low-energy flux arriving under glancing angles, leading to films consisting of areas with dense columns perpendicular to the substrate and open-voided inclined columns (compare Figure 1a,b) [19].

There is a recent trend to add more and more functionalities to thin metallic films, for example, to achieve a suitable combination of oxidation resistance with electrical and optical properties, demanding complex film alloy designs with multiple elements [27]. While in lab-scale experiments combinatorial sputtering from multiple magnetrons using different single-element targets is widely used to identify “sweet spots” in the chemical composition [28], large-area deposition necessitates sputtering from multiple-element targets. There, translating the target composition directly into the thin film composition is often challenging due to selective sputtering and gas phase scattering of the individual elements [19]. As an example, both films presented in Figure 1a and b are sputtered from the same rotatable Mo_0.70_Al_0.20_Ti_0.10_ target in the same deposition system using the same process parameters. The film deposited directly in front of the target (Figure 1a) has a chemical composition of Mo_0.72_Al_0.17_Ti_0.11_, it undergoes loss of Al due to the enhanced scattering of light Al atoms colliding with the Ar process gas. For the film shown in Figure 1b, which was grown under inclined conditions to simulate substrate oscillation with a factor of 2.7 higher target-substrate distance, a composition of Mo_0.67_Al_0.23_Ti_0.10_ is obtained, indicating that for these high distances the pronounced scattering of Mo atoms also takes place [19]. As a consequence, metal films composed of multiple elements, even when sputtered from multiple-element targets, will have variations in morphology and chemical compositions when grown on oscillating substrates. The deposition of high-quality metal films on flexible polymer substrates poses some more challenges. While in many research studies polyimide (PI) substrates with their high thermal stability of up to more than 300 °C have been used, polyesters such as polyethylene terephthalate (PET) or polyethylene naphthalate (PEN) show advantages with respect to optical clarity, but suffer from low glass transition temperatures of 80 or 150 °C, respectively [29]. The softening of polymers at elevated temperatures may also lead to the release of gas trapped in the polymer, which may deteriorate film adhesion [30]. This means that thermal overloads of the substrates, by heat radiation from the magnetron plasma running at high power or by energetic particle bombardment of the substrate, need to be avoided. In a recent study, Jörg et al. have shown that sputtering a single rotating cylindrical molybdenum target with dimension ∅ 150 mm × 600 mm at 10 kW d.c. power under close to industrial conditions results in a rise of the substrate temperature up to 300 °C within 250 s [31]. On a more local scale, the bombardment of the polymer surface during substrate pre-treatment or in the early film growth stage with energetic species may alter the near surface zone of the polymer, thus affecting adhesion [32]. An example for the near surface zone of a PI substrate modified by thermal vapor deposition of an Al film is shown in Figure 1c, where clearly a few nanometers-thick interlayer is visible. Tailoring of such interlayers might potentially be used to enhance adhesion. Consequently, such a flexible thin film system should be treated as composed of three layers, the polymer substrate, the interface zone, and finally the film itself.

Avoiding overheating of thermally sensitive substrates means that film growth occurs with low thermal activation, leading to a dominating influence of atomic shadowing on the growth of the nucleated islands (see Section 2.3). Under these growth conditions, deposition of dense films with the typical low thicknesses of a few 10 nm, e.g., for interconnect lines, diffusion barriers, or adhesion layers, is challenging.

Another limitation in film growth stems from the low electrical conductivity or even insulating nature of the polymer substrates. In many cases, large-area sputter deposition is performed using grounded substrate holders that do not have the possibility to apply a negative substrate bias voltage to kinetically activate film growth. Subjecting the polymer substrate to the plasma means that its surface is exposed to a zero-net flux of ions and electrons, where—as a result of the different ability to be accelerated within the electrical field—charging of the polymer surface up to the negative *floating potential* (see Section 2.1) occurs. The proceeding growth and coalescence of an electrically conductive film forming islands then leads to the de-charging of the surface, changing the conditions of bombardment of the growing film with charged species. On a large-area substrate oscillating in front of the plasma of the magnetrons, local charging and de-charging effects will lead to changing conditions during the early film growth stage. 

Finally, sputter deposited films are typically under a tensile or compressive stress state stemming from thermal (which are assumed to be low because of the necessary low substrate temperatures), intrinsic (stemming from growth defects), and extrinsic contributions (originating from structural misfit, phase transformation, precipitation, plastic or creep deformation, chemical reactions, etc.) [33]. Tensile stresses exceeding the cohesive energy of the film will result in crack formation, whereas compressive stresses may lead to spontaneous delamination buckling, provided that the film adhesion is sufficiently low [34]. Careful optimization of the deposition conditions for stress minimization is necessary to avoid the unacceptable deterioration of the film/polymer system.

### 2.5. Other Thin Film Deposition Methods

Despite the challenges associated with the sputter deposition of thin films onto polymer substrates summarized in Section 2.4 and high investment costs of vacuum deposition systems, the scalability from lab-scale research to high-throughput large-area deposition, excellent process controllability, and the versatility in terms of available film materials and their microstructures combined with the ability to grow high-quality films at temperatures compatible with thermally sensitive substrate materials make this technique unique [18]. Besides vapor deposition, other processes using liquid precursors are used to coat polymer substrates such as spray-, dip-, and spin-coating. These techniques are popular in research, due to their low investment costs and easy process optimization, but some of them are inherently nonuniform over large areas, which poses challenges on scalability [35]. Among these techniques, dip-coating is suitable for roll-to-roll coating, enabling high-throughput film deposition on thin flexible polymers [36].

Inkjet printing represents an attractive technique for patterning, or the “direct writing” of well-defined thin film metal lines with widths down to the micrometer range, thus avoiding masks [37]. A drawback of such solution-based methods is the use of solvents carrying metallic nanoparticles and binders [38], or self-reducing methods [39], which necessitate post-deposition curing to remove solvents and to sinter the nanoparticles. Even then, films are significantly less dense than sputter deposited films, as evidenced by the SEM cross-section of a printed Cu film in Figure 1d. Consequently, the electrical conductivity of these printed lines is well below that of the corresponding bulk metal [40].

The various deposition methods and parameters provide a wealth of opportunities to tailor thin metal films. After deposition, the flexible metallic films must be properly characterized electrically and mechanically. Such characterization techniques are relatively new to the field. The subsequent sections provide an introduction into the electro-mechanical testing of thin metal films deposited onto flexible polymer substrates (Section 3) and a discussion of some thin film material design aspects based on metallic thin film deposition methods to optimize their performance for flexible electronic applications (Section 4). In agreement with the importance of sputter deposition processes, most of the examples chosen will be based on sputter deposited metal films.

## 3. Electro-Mechanical Testing

The most efficient way to examine the electrical and mechanical behavior of flexible metal film systems is to use uniaxial tensile straining methods. The tensile straining of metal or ceramic/oxide films on polymer substrates can also be referred to as fragmentation testing or periodic cracking and was initially used in composites and ceramic films on metal substrates [41,42,43]. With the introduction of flexible electronics at the turn of the 21st century, the same technique was applied to metal-polymer and ceramic-polymer systems [44,45,46,47,48]. The main concept behind fragmentation testing is the shear lag model [41,42,49] that can be used to determine the interfacial shear stress, *τ_IFSS_*, to quantify the stress the interface can carry (Equation (1)):

*τ_IFSS_* = *π h_f_ σ_frac_*/2*λ_sat_*. (1)

**Figure 2:**
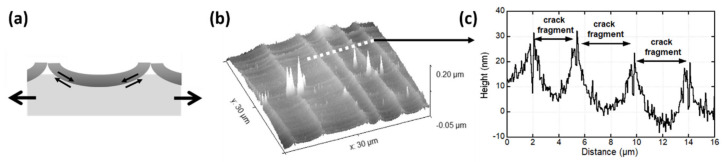
(**a**) Schematic diagram of fragments bending in cross-section, (**b**) 3D atomic force microscopy height image of crack fragments in a 200 nm sputter deposited Cr film on PI at 10% strain. White dashed line indicates the profile in (**c**).

In Equation (1), *σ_frac_* is the initial fracture stress of the film, *λ_sat_* is the average linear crack spacing at saturation (when no further cracks form), and *h_f_* is the film thickness. The initial fracture stress is best measured with X-ray diffraction (XRD); however, many groups have also used Hooke’s law and the observed fracture strain (i.e., *σ_frac_ = E_f ·_ ε_frac_* with *ε_frac_* the observed fracture strain, and *E_f_* the elastic modulus of the film). An important aspect to remember is that in order to apply the shear lag model, the ratio between the maximum and minimum crack spacings at saturation must be two. Only then is the shear lag model applicable [41,49]. Small changes in the film thickness or large surface roughness have been shown to alter this ratio for brittle films on metal or polymer substrates [50,51]. In these cases, the neighbor ratio can be applied [50,52]. The neighbor ratio evaluates the crack spacing on either side of a crack. With a neighbor ratio value around two and lower, the film/polymer system can be evaluated with the shear lag model and values much higher than two indicate that the shear lag model cannot be used. In the cases it cannot be used, the most common reason is that the film fragments on the polymer substrate do not remain parallel to the straining direction. Instead, the fragments bend into the more compliant polymer substrate [51,53] (Figure 2) and cause an inhomogeneous stress transfer at the interface. Substrates with low elastic modulus values, such as PET or PEN, are more likely to have significant fragment bending compared to other substrates with higher elastic moduli, such as PI which ranges from 3 to 9 GPa.

### 3.1. Brittle Film Behavior

**Figure 3:**
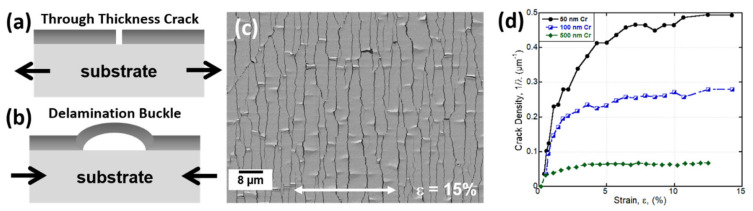
Schematic diagram of (**a**) through thickness cracks (channel cracks) that form perpendicular to the straining direction and (**b**) delamination buckles which form parallel to the straining direction. (**c**) SEM micrograph of a 100 nm sputter deposited Cr film strained to 15% to illustrate cracking and delamination of the film. White arrow indicates the straining direction. (**d**) Crack density evolution for three sputter deposited Cr films (50, 100, 500 nm) on PI illustrating the effect of film thickness.

The tensile straining of metal films on polymer substrates can be straightforward depending on the type of film system being examined. Films or layers that behave in a brittle manner are the easiest film systems to interpret because only two things can occur: (1) fracture followed by (2) delamination (Figure 3a–c). Brittle films, generally body-centered cubic (BCC) or hexagonal-closed packed (HCP) metals, such as single layers of Cr, Mo, Ti, Ta, along with indium tin oxide and other oxides [31,51,52,54,55,56,57,58,59], and even metallic multilayers and printed films [60,61,62,63,64,65,66,67] will easily fracture at low applied strains. With in-situ or intermittent imaging of strained samples using optical light microscopy, SEM, or confocal laser scanning microscopy (CLSM), the fractures of films in the form of through thickness cracks (TTCs), also called channel cracks, are observed as well as delamination buckles that form between the crack fragments. At low applied strains (<1–2%), cracks will initially form perpendicular to the straining direction. The strain at which cracks are first observed to form is referred to as the crack onset strain (COS) or the fracture strain, *ε_frac_*. Cracks will continue to form with continued straining until a saturation crack spacing, *λ_sat_*, or saturation crack density, 1/*λ_sat_*, is reached (Figure 3d). In general, thinner films have higher crack densities (smaller crack spacings) and thicker films have lower crack densities (larger crack spacings). Delamination occurs at much higher applied strains (approximately 8–12% strain, depending on the film thickness and substrate) usually after the film fragmentation (cracking) has reached a saturation [51,68,69]. Delaminated areas, also called buckles, of the film are observed between crack fragments parallel to the straining direction and form because a compressive stress high enough to cause delamination is created due to the difference in Poisson’s ratio between film and substrate under uniaxial tensile straining. These buckles can be used to quantify the adhesion energy of the metal–polymer interface [55].

### 3.2. Ductile Film Behavior

**Figure 4:**
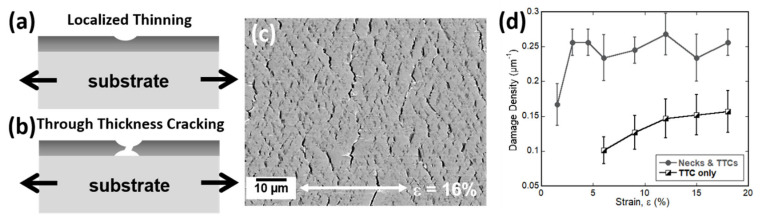
Schematic diagram of (**a**) localized thinning (also called necking) that first forms when a ductile metal film is strained. With continued straining (**b**) many of these areas will become TTCs. (**c**) SEM micrograph of 200 nm electron beam evaporated Cu film strained to 16% to illustrate localized necking and TTC formation (black lines) of the film. White arrow indicates the straining direction. (**d**) TTC and damage evolution of a 200 nm Cu/10 nm Cr bilayer film system roll-to-roll sputter deposited on PET. The combined damage density of necks and TTC spacing is higher and starts before TTC cracks form.

Compared to the brittle film systems that easily fracture and delaminate, ductile film systems will undergo plastic deformation before cracks form. Ductile metals, such as face-centered cubic (FCC) Au, Ag, Al, or Cu, are typically used as conductors in flexible electronics or as reflectors in multilayer insulators and optical solar reflectors. Uniaxial straining of single ductile metal layers has a different set of mechanisms than brittle metal films. The main difference is that ductile films can plastically deform more than their brittle counterparts. Therefore, instead of observing the fracture at low applied strains, a deformation onset strain (DOS or *ε_def_*) is observed and corresponds to the initial necking, or localized deformation, of the film (Figure 4a–c). With further applied strain, the necks can become TTCs; however, not all necks will become TTCs [70]. In single ductile films, necking will initiate at the surface. In-situ straining of single ductile layers is best performed with a 3D surface imaging method, such as atomic force microscopy (AFM) or CLSM. These two methods allow for the determination of the neck or TTC depth [70,71].

For single layer ductile films, the deformation is observed to start at the surface. Therefore, AFM and CLSM are excellent imaging techniques to determine the depth of the necks or TTCs using the Δ/*h* ratio, where Δ is the depth of the damage (neck or TTC), and *h* is the film thickness. In AFM and CLSM, the probe form might limit access to the deepest part of the crack [72]; therefore, a neck is defined when the Δ/*h* ratio is less than 10–15% of the film thickness and a TTC when the Δ/*h* ratio is greater than 10–15% of the film thickness, depending on the surface roughness of the film [70,71,73,74]. The evolution of the ductile deformation, also called damage, of a film can be quantified similar to crack spacing (Figure 3d). Damage always starts first, followed by TTC formation (Figure 4d). The damage density will be higher than the TTC density because the damage density includes both necking and TTC sites, while TTC density is only the density of TTCs. Additionally, with in-situ AFM or CLSM the TTC closure, when the tensile load is removed, it can be quantified [73].

### 3.3. Electrical Behavior

**Figure 5:**
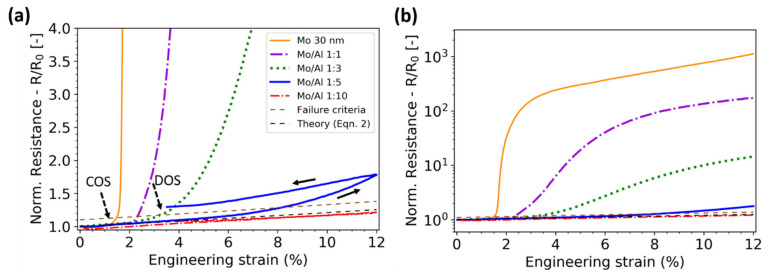
(**a**) Example of brittle and ductile electrical behavior in sputter deposited Mo and Mo/Al bilayers with increasing Al thickness (Mo:Al layer thickness ratio) as a function of applied strain. Deviation from the constant volume approximation generally is referred to as the COS or DOS. Note that the more ductile bilayers have a variety of responses compared to brittle 30 nm Mo film. (**b**) At high applied strains, the resistance does not reach a distinct plateau even though the crack or damage density saturates (note log scale). Only loading data shown for clarity. Data taken from [80].

For flexible electronics, the electrical behavior needs to be measured as a function of applied strain. In-situ resistance measurements are the optimum method to measure the electrical response as a function of applied strain. Lu, Wang, and Vlassak [75] introduced the constant volume approximation as a way to determine the COS of metal or conductive ceramics supported by polymer substrates. The constant volume approximation evaluates the electrical properties of a material as a function of strain, as follows (Equation (2)):

*R/R*_0_ = (*L*/*L*_0_)^2^ = (1 + *ε*)^2^, (2)

where *R* is the measured resistance, *R*_0_ the initial resistance before straining, *L* the gauge length, *L*_0_ the initial gauge length, and *ε* the strain [75]. Any deviation from the constant volume approximation is related to changes in the material initiating from the geometrical changes due to straining [76]. This could be an increase or decrease caused by defects, such as TTCs, grain growth, high residual stress, or phase changes. From the constant volume approximation, the formation of structural damage, such as TTCs, can be indicated. For brittle films a strong and sudden increase in *R*/*R*_0_ or Δ*R*/*R*_0_ signals that at least one TTC is long enough to hinder the electric current flow (Figure 5a). Many researchers have reported that the sudden increase in resistance is correlated to the observed crack formation in brittle films [77,78,79,80,81,82,83]. The increase in the electrical resistance of ductile film systems, on the other hand, does not always signal a fracture. DOS occurs somewhere between necking and TTCs’ formation because TTCs are much shorter in ductile films and necks can still conduct currents. In the case of ductile film systems, a 10–20% deviation from the constant volume approximation has been used as a failure criterion [80]. This criterion avoids any opinions as to how “an increase” from the constant volume approximation could be defined (Figure 5a). In Figure 5, the electrical response of a single 30 nm Mo film is compared to Mo/Al bilayers with various Al thicknesses (thickness ratios of 1:1, 1:3, 1:5, and 1:10, with a 30 nm Mo constant) to illustrate different responses due to ductile Al film thickness. 

The electrical behavior is considered to be a global measurement, considering ALL necks and TTCs that form between the contact grips (or contacts) used for straining. In-situ AFM, CLSM, and even XRD experiments have a finite, or local, area that is observed or measured during straining. Thus, the local measurements may not tell the whole story of the global electrical response. What the electrical DOS indicates is that a critical density of structural defects is present at a specific applied strain that increases the electrical resistance between the electrical contacts, rather than a distinct COS. The structural defects could be a few long TTCs, multiple short TTCs, or more severe pre-existing defects such as substrate scratches. It is important to understand that while the damage density (TTCs and/or necks) will saturate (Figure 3d and Figure 4d), the electrical resistance does not (Figure 5b). It is also beneficial to measure the resistance during loading and unloading of the experiment (Figure 5a, solid blue line, Mo/Al 1:5, with arrows). As the sample is unloaded (downward arrow), the viscoelastic recovery of the substrate occurs and TTCs in the metal film can re-bridge, or close, and help to reduce the measured resistance. FIB cross-sectioning has provided evidence to the crack re-bridging, for example Figure 5 in [76]. Additionally, an in-situ experiment with AFM and electrical resistance by Cordill et al. [73] quantified the resistance recovery and related it to the decrease in TTCs’ spacing and increase in deformation (necks and TTCs) spacing. In the early literature, which utilized the in-situ resistance measurement, the maximum resistance at the maximum applied strain was reported with an image of an unloaded film at a different unloaded and recovered strain. Thus, the reported resistance did not relate to the damage or TTC density in the provided image since a portion of the TTCs closed and were no longer visible as TTCs. 

A recent development to better understand resistance recovery and crack re-bridging is to use the cracking factor [84,85]. This approach uses the electrical resistance and the crack density to estimate an effective crack length, *ℓ_eff_*, of the TTCs travelling along the plane of the film. Additionally, the electro-mechanical behavior can be expressed by a simple quadratic expression (Equation (3))

*R/R*_0_ = 1 + (1/√2)*C_l_ℓ_eff_* + (1/2)*C_l_*^2^*ℓ_eff_*^2^, (3)

where *R*/*R*_0_ is the normalized resistance, *C_l_* is the linear crack density, and *ℓ_eff_* the effective crack length. There are limitations to the cracking factor approach [84]; however, in general it can be applied to ductile films exhibiting short cracks. The model assumes all cracks have the same length, while in reality there is a distribution of short and long cracks. More research in this area is needed in order to fully understand the information in-situ resistance measurements provide other than a COS or DOS.

**Figure 6:**
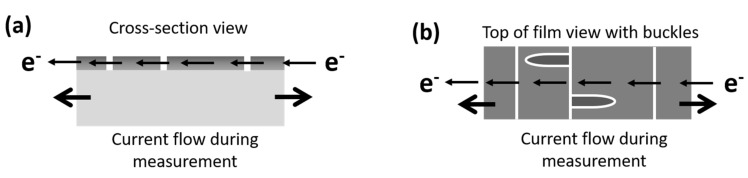
(**a**) Electrical resistance measurements are performed perpendicular to the TTCs. (**b**) If the film forms buckles, the measured resistance will not be influenced because buckles are parallel to the measurement direction. Resistance cannot be used to detect delamination.

It must be noted that in-situ electrical resistance measurements do not provide information about delamination or film buckling. The resistance is measured parallel to the interface that is delaminating (Figure 6). If a buckle forms, the film is still conductive (and more conductive than the polymer substrate). When it is not connected to the substrate there would be no significant change in the measured electrical resistance as it is nearly impossible to decouple the resistance contributions of cracks and buckles in the crack saturation regime. The current flow is also parallel to the direction of buckle formation and would not influence the measurement. Only cracks perpendicular to the measurement are detected. Furthermore, when buckling does occur, it is at high applied strains where the electrical resistance is already quite high due to the presence of TTCs. No additional information is gained at these high applied strains and resistances (Figure 5b).

### 3.4. Cyclic Testing

**Figure 7:**
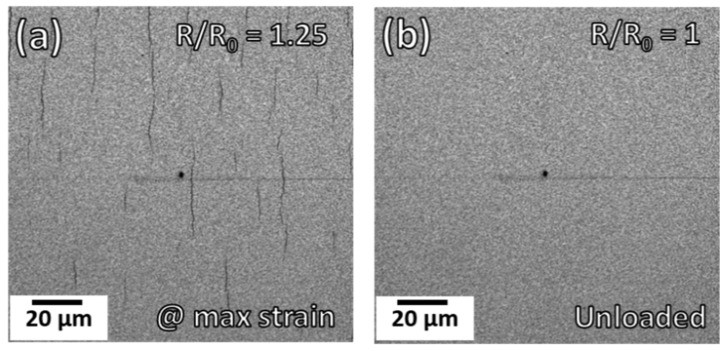
CLSM laser intensity images of a printed Ag film at (**a**) a maximum applied strain of 2% having open cracks (black lines) with *R*/*R*_0_ = 1.25. (**b**) Removing the load returns the *R*/*R*_0_ back to its original value of 1 and cracks are no longer visible. (Images courtesy of O. Glushko.).

The cyclic mechanical testing of flexible thin films can be performed in different ways, including uniaxial straining [79,85,86,87,88,89,90,91,92] and bending [93,94,95,96,97,98,99,100,101]. The uniaxial approach is the more simple experiment to perform and the resistance can be easily added for in-situ measurements [87,102]. It should be noted that with bending, the bending is often applied differently, on a cantilever [93,99,103], in shear creating two zones of damage [99,101,104], at a right angle (90°) to generate one zone of damage [97,98], as well as other configurations [105,106] which makes comparing results between the different methods a challenge. What is known about cyclic loading with in-situ resistance measurements is that when cracks open the resistance increases, and when cracks close the resistance decreases [66,107] (Figure 7). The evolution as a function of the cycle number of the maximum *R*/*R*_0_ and minimum *R/R*_0_ are different for different materials, fabrication methods, microstructures, and loading (uniaxial vs. bending). For most metallic film systems, the Δ*R = (R_max_* − *R_min_)* during cycling tends to be small until TTCs form, which significantly increase the Δ*R*. Exemplary papers on in-situ cyclic tensile testing with resistance will provide more details [66,85,86,87] including using the cracking factor [85]. For bending, in-situ and intermittent testing methods have been used [94,95,97,104,108]. An important concept to know is that measuring the COS or DOS with uniaxial straining and remaining below that value for cycling under bending will not necessarily lead to an unbreakable film system [108,109]. Only testing for the real application, stretching, or bending, will provide the proper material failure information.

While more is known and understood about how thin metal films on polymer substrates fail under monotonic tensile straining, the field still has a long way to go (Table 1). Crack formation causing electrical failure is clear and the failure can be quantified with a crack spacing or damage density. However, cyclic tensile straining and cyclic bending are areas that need a clear definition of failure to precisely determine reliability. Is it when the electrical resistance increases by 10% or 20% or when a certain value for the electrode resistivity is exceeded? Is it when the first TTC is observed, or, does the saturation damage or crack density define the lifetime? What about the difference between sensing applications and devices? Do these different applications require different failure criteria? These questions can only be answered with the direct collaboration of universities/research institutions with the industry and the full understanding of how mechanical film damage influences the electrical behavior (summarized in Table 1).

## 4. Material Influences

With an understanding of how flexible metallic thin film system are evaluated, material design approaches can be applied. In this section, various thin film and material engineering concepts are described that improve the COS, DOS, or cyclic lifetime (as defined as a 20% increase in relative resistance) of metal films on polymer substrates. Simple but elegant approaches are illustrated that positively (or negatively) influence the electro-mechanical behavior as well as customizing the film architecture or chemistry. These approaches include film thickness, residual stress, and microstructural tailoring and will be discussed in detail.

### 4.1. Improving Fracture Resistance of Brittle Films

The simplest way to alter the COS of a brittle metal film system is to decrease its film thickness. As demonstrated earlier with the use of fragmentation testing, film thickness is directly related to the crack spacing or crack density [41,50,52,54,68,110,111,112]. A thick film will have a large crack spacing (low crack density) while thin films have a small crack spacing, or large crack density (Figure 3d). The behavior is simply due to the amount of stress a film fragment can carry before reaching the film’s fracture stress and is described by the shear lag model (Equation (1)). Along these lines, thinner films will reach higher fracture strains, or COS, compared to the same film with a larger film thickness, regardless of the deposition method. Some good examples in the literature are [51,52,54,68,83,110].

Initially, the role of a film’s residual stress was overlooked, and systematic studies were not performed for the same film or film thickness. Now, it is known that with control over the deposition parameters, the residual stress of metals’ films can be controlled very well, and even tailored for a specific application. Jörg et al. [83] demonstrated for sputter deposited Mo films with thicknesses between 50 nm and 500 nm, that a high compressive stress could improve the COS by a factor of three for the same film thickness when strained with uniaxial tension. Additionally, the film residual stresses could be highly tensile to highly compressive with only changing the target power or Ar pressure during magnetron sputtering [31]. The residual stresses were measured with XRD on Si and PI substrates and with a curvature-based method [113]. Further work on tailoring the stresses of Mo films revealed that even under cyclic tensile loading an improved lifetime was observed [102]. The opposite stress tailoring could also be used when the application calls for compressive loading, such as with bending. In this case, a tensile residual stress would be ideal to achieve a high COS and ideally more cycles to failure. Therefore, for a brittle metal film, a thickness of about 10 nm with a high compressive stress is desired for stretchable applications, and a high tensile stress for compressive bending applications.

**Figure 8:**
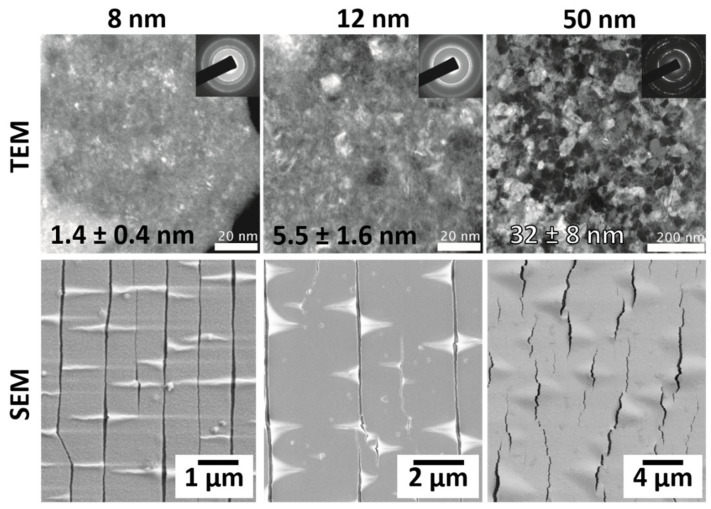
Electron beam evaporated Ti films (8, 12 and 50 nm thick) on PI have a transition in the crack morphology from long straight cracks to wavy, zig-zag cracks (bottom row) due to the increase in grain size (numbers in images) with increasing film thickness (top row) [52]. TEM images reprinted from Thin Solid Films, Vol. 589, M.J. Cordill and A.A. Taylor, Thickness effect on the fracture and delamination of titanium films, pp. 209–214, (2015) with permission from Elsevier.

For brittle films, the morphology, or shape, the crack takes is mostly dependent on the film microstructure. Cracks in ceramic and oxide films, such as indium tin oxide (ITO), will generally be very long and straight, propagating quickly across the whole sample width due to a lack of crystallinity (amorphous) or very small grains [68,79]. Metal films, however, can have straight or wavy (also called zig-zag) cracks that also quickly propagate across the whole sample width. The crack shape is dependent on the grain size and residual stress of the film, which is determined by the deposition method and used parameters. Brittle metals tend to have small grain sizes because they are often very thin when used as adhesion interlayers. A general rule of thumb is that the grain size of a thin film is on the order of the film thickness [114], thus, a 50 nm thick film would have an average grain size of tens of nm. In most cases, the grain morphology of a thin film is columnar (see Figure 1a,b). When strained, cracks will propagate along the weakest, or more energetically favorable, paths, which tend to be along the column or grain boundaries. With very small grains, the cracks appear to be straight. The appearance of wavy cracks in single phase and single layer films is due to a larger grain size and the connection of smaller cracks. An example of how the film thickness, microstructure, and crack morphology are related can be found in Figure 8 for three thicknesses of electron beam evaporated Ti films on PI [52]. There is a clear transition in grain size and crack morphology from the 8 nm film with straight cracks, mixed cracking in the 12 nm film, to full wavy cracks in the 50 nm film. In alloy or two-phase films, discussed later, the crack morphology is also related to the grain size, phase distribution, and chemistry. Cracks will still tend to form along column or grain boundaries, but the propagation of the cracks will be more tortuous as the path must go around the different phases to find the more energetically favorable path [115,116,117]. Highly stressed brittle films, for example, films with a high tensile residual stress, can be pre-cracked. Therefore, additional cracking due to straining will connect pre-existing cracks with newly formed cracks. It is a common misconception to describe wavy or zig-zag cracks as an indication of more ductility in the films when the films illustrate brittle fracture. Ductility can only be measured with a stress–strain curve and not by the crack morphology. The wavy cracks are a direct result of shorter TTCs initiating at low applied strains due to the difference in grain size or phases and connecting at higher strains. The films should still be considered brittle as TTCs form easily when strained or bent. Ductile films, as presented in the next section, fail via different mechanisms than brittle films, thus, the crack morphology for brittle films being straight or wavy is generally unimportant. Rather, as previously discussed, the effective crack lengths of the TTCs are important for the combined electro-mechanical behavior.

### 4.2. Microstructural Influences

Similar to varying the residual stress of a film, the microstructure can also be tailored by changing the deposition parameters. When deposited, some metals will form a bi-modal microstructure with small grains at the interface and large grains as the film thickness increases due to island growth of the film [83,118]. With the right deposition parameters and post-deposition heat treatments, the microstructure can be directed to provide the desired mechanical and electrical properties. 

Often brittle metal films, such as Cr, Mo, and Ta, tend to have small grain sizes due to the small thicknesses. Sophisticated deposition conditions, such as oscillating the substrate carrier in front of the target during deposition to create a zig-zag column structure [102], is a way to further alter the microstructure of a thin film. The zig-zag structure was shown to be crack arresting because it can stop long cracks from forming in the plane of the film [102]. Additionally, the zig-zag structure is just as effective under monotonic straining conditions as residual stress tuning for columnar microstructures and even more effective under cyclic tensile straining when the residual stress is also tailored [102]. For the zig-zag microstructure, cracks must continuously change direction to propagate through the film thickness as well as along the plane of the film (across the sample). As such changes in direction need to overcome energetical barriers, the cracks actually stop propagating. Self-reducing printed Ag films can also have a self-arresting structure created from the inherent porous structure of the films [39]. A similar method to arrest cracks is to deposit a film with holes using photolithography methods, also known as micro-patterning. It was shown by the authors in [119] that the presence of holes causes cracks to blunt quickly rather than travel across the width of the thermally evaporated 500 nm thick Ti film on PI. While this improvement is not necessarily due to the film microstructure, the film geometry (blanket vs. with holes) is altered to have crack arresting abilities. However, it should be mentioned that the crack resistance came at the cost of lower conductivity for both the zig-zag and hole structures.

Room temperature phase changes can also be used to improve the mechanical performance of metal films on polymer substrates. An example is the martensitic phase transformation in 2 µm thick sputter deposited Co films on PI from a metastable (002)_fcc_ to stable (10–10)_hcp_ and (10–11)_hcp_ [120]. Using in-situ XRD straining experiments with resistance measurements, a reduction of the FCC phase started at less than 1% applied strain and was observed before TTCs formed at about 6.7% applied strain. The phase transformation was aided by the formation of necks between applied strains of 2% and 6.7%. Crack saturation was achieved after 10% applied strain with the phase transformation ending after applying 8% strain [120]. In another study, annealing two-phase Co films prior to straining significantly increased the FCC phase. The annealing also led to the higher COS for films between 200 nm and 2 µm thickness, with increased necking deformation before TTC formation for thicker films compared to thinner films [121]. Therefore, room temperature phase transformations are another efficient way to increase the fracture resistance of metal films.

For a ductile film system, Cu films are often studied because the microstructure can be controlled not only with deposition parameters, but also with post-deposition annealing treatments. As shown by Berger et al. [74], a 200 nm thick Cu film on PI initially had an in-plane bi-modal grain size and after annealing for 2 h at 200 °C in a vacuum (without removing from deposition chamber), a homogenous grain size of about 2 µm was observed. Monotonic straining with in-situ electrical resistance, XRD, and AFM found clear differences between the electro-mechanical behaviors of the annealed and as-deposited films. The maximum stress the films reached was directly related to the film structure with the annealed film having a lower maximum stress. The saturation deformation spacings were similar; however, TTCs were observed in the annealed film and not in the as-deposited film. Cracks and necks will form initially in large grains where it is easier for dislocations to glide, especially when twin boundaries are present. The annealed film’s electrical response (*R*/*R*_0_) was always above the as-deposited film. This electrical behavior indicates that the bi-modal microstructure actually is more resistant to damage (less resistance increase) than the annealed film with larger grains and fewer grain boundaries.

When these same and similar Cu films were subjected to cyclic tensile straining, a more significant difference in electro-mechanical behavior was observed. With cyclic loading, a resistance decrease was found for Cu films with a bi-modal grain size distribution [122]. This improved resistance has been directly related to grain growth [87,122] and has also been observed in small-grained Au films [86,123] and could be used as a material design element. Ductile metal films with larger grains will not have any grain growth as the structure is already stable. Therefore, deformation and eventual TTCs will more likely form earlier compared to films with a bi-modal grain size distribution or small grains. In general, under monotonic or cyclic tensile loading, small grains in ductile films equal to or thicker than 200 nm are preferred to large homogenously sized grains. In thinner ductile films, small grains can lead to an immediate fracture [64].

### 4.3. Film Architecture and Thickness Ratios

The previous sections considered single layer films; however, most flexible thin film systems consist of two or more layers. For this discussion, multiple layered films and the layer order will be referred to as the film system’s *architecture*. The type of material (FCC metal—ductile/D, BCC metal—brittle/B), the thickness of the layers, or thickness ratio, and the order of the ductile and brittle layers (layer order) will be examined. Focus will also be on metallic, crystalline thin films, rather than ceramic or glass film systems.

Since the early development of metal films on polymer substrates, brittle metal adhesion layers have been used [47,48,75,124,125,126]. The initial reason behind using the additional layers was to improve adhesion of the ductile conductive layer as poor adhesion was assumed to be the underlying mechanism of crack formation in ductile films [127,128], and for protection due to certain oxidizing processing steps during manufacturing [128]. Since these early works, more has become known about how ductile films on polymer substrates fail mechanically and adhesion does not have as large as an influence as initially believed. Furthermore, with proper deposition parameters, ductile films like Cu, Au, Ag, and Al can have very good adhesion to polymer substrates without using brittle adhesion interlayers [32,64,65]. What has been demonstrated with in-situ XRD, AFM, CLSM, and resistance measurements is brittle interlayers (D/B) and top layers (B/D) cause the ductile films to fail much sooner than if the brittle layer was not present. This behavior has implications not only for flexible electronics and sensors, but also for nanoscale multilayers [60,61,129] and thin films used in space applications [65,130,131].

**Figure 9:**
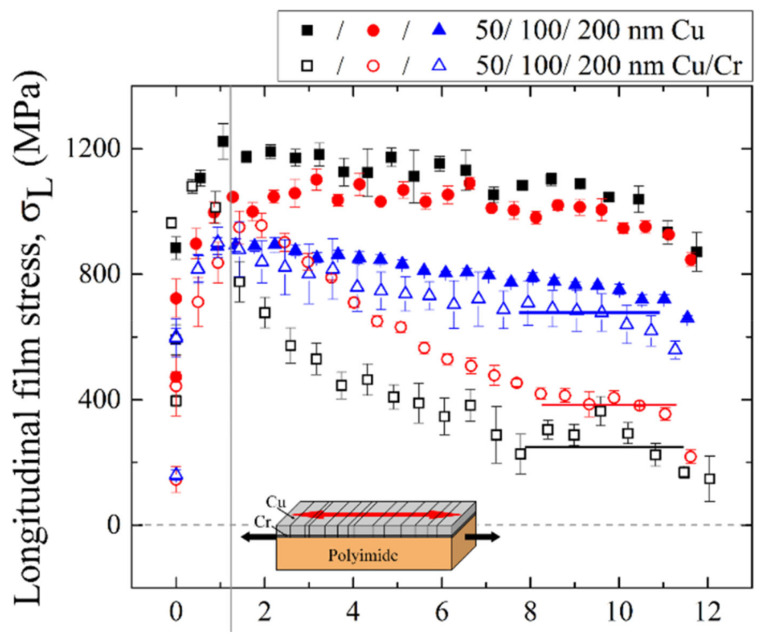
In-situ XRD stress measurements during tensile straining of single layer sputtered Cu films (50, 100, 200 nm, filled symbols) and Cu/Cr bilayers (10 nm Cr interlayers, same Cu thicknesses, open symbols) illustrate that when brittle interlayers are used, the Cu film stresses decrease after reaching a maximum stress due to TTC formation. The single Cu layers continue to have high stresses with further applied strain because TTCs do not form. Figure from [64]. Reprinted from Acta Materilia, Vol. 89, V.M Marx, F. Toth, A. Wiesinger, J. Berger, C. Kirchlechner, M.J. Cordill, F.D. Fischer, F.G. Rammerstorfer, G. Dehm, The influence of a brittle Cr interlayer on the deformation behavior of thin Cu films on flexible substrates: Experiment and model, p. 278–289, (2015) with permission from Elsevier (CC by 4.0).

Several studies have demonstrated that brittle interlayers cause the early electrical and mechanical failure of ductile films. From experiments and simulations [64], the early failure of normally ductile films with brittle interlayers, such as Cr, Ti, or Mo [64,67,80,122,132,133], has been observed, again independent of the deposition method. In-situ resistance measurements note a deviation from the constant volume approximation well before that of the same film without the interlayer and are dependent on the ductile film thickness [80]. The most telling in-situ experiment is with XRD. Marx et al. [64] demonstrated that the Cu film in Cu/Cr bilayers would have a stress–strain curve shape similar to brittle Cr (Figure 9). In the Cu/Cr bilayer, the Cu stress increases to a maximum value then decreases, thus, losing the ability to carry stress because TTCs have formed (Figure 9, open symbols). The single Cu layers (Figure 9, filled symbols) also achieve a maximum stress, but the stress remains high without decreasing with increasing applied strain, which indicates that the film has not formed TTCs. Finite element simulations, performed to better understand these experiments, found that the thin 10 nm Cr layer fractures at about or below 1% applied strain, creating cracks [64]. The Cr cracks act as stress concentration points, causing necking and TTC formation in the Cu films at these critical points. More recent work on sputter deposited Au/Cr [78] and Cu/Mo [133] with additional in-situ resistance measurements have determined that the maximum stresses are related to the fracture stress of the films.

More experiments were performed with sputter deposited Cu and Mo bilayers with different film thickness ratios and with a slightly thicker Mo film. With the thicker Mo film, not only could the Cu stress be measured, but also the stress of the Mo layers [133]. With the mechanical information of both layers, it was found that the thicker Cu layers can actually improve the apparent fracture toughness, *K_Ic_*, of the Mo layer, up to a thickness ratio of 5:1 (Cu:Mo). Similar research on sputtered Al/Mo bilayers strained biaxially has also demonstrated an improvement in the fracture stress of the Mo interlayer with thicker Al layers [118,132]. When the bilayer is reversed to Mo/Al, the opposite trend is observed, and thinner Al films improve the fracture strain of the Mo film [118].

It should be noted that the thickness ratio between the ductile layer and brittle layer can be used as a design criterion, but it is not universal and is different for every material system. Under monotonic tensile straining in order to suppress TTC formation in the ductile layer, a thickness ratio of 20:1 is best for the Cu/Cr on PI [64], and for Al/Mo on PI a thickness ratio of 10:1 works well [80]. However, for Au/Cr also on PI, 5:1 is not enough to hinder TTC formation, especially when the films are 50 nm and 10 nm, respectively [67,78]. The Inconel-Ag on Teflon [65] with a thickness ratio of 1:5 is also not sufficient with thicknesses of 30 nm and 150 nm (Ag) and the reversed layer order (brittle layer on top). Further studies, especially with in-situ XRD, are necessary. XRD is the only technique that can probe multiple material behaviors simultaneously, and thus is the only method suitable for bilayer and multilayer behavior to be properly investigated. These initial results strongly indicate that the film architecture matters, and once understood, could be exploited as a powerful material design concept. 

### 4.4. Alloying

**Figure 10:**
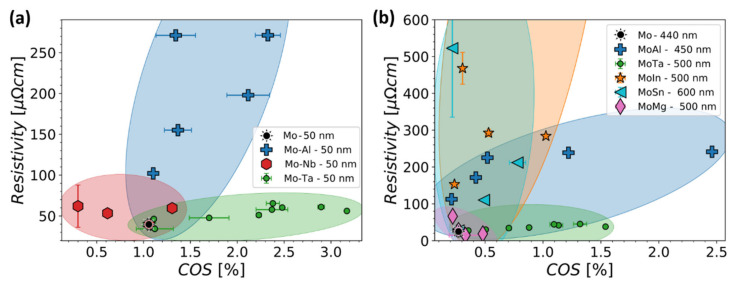
Map of electrical resistivity versus COS for (**a**) 50 nm and (**b**) 500 nm thick Mo-based alloy film systems with different chemical compositions. The highlighted areas act as guides for the eye to collect similar alloy systems and have no mathematical or statistical meaning.

Thin film deposition techniques allow for a variety of metal alloys to be achieved, even if they are unstable in the bulk form. In the research stage, co-sputtering with multiple targets permits for a wide range of chemistries and structures to be created and evaluated as thin films. Alloying is an excellent technique to increase fracture resistance in brittle metals films [115,116,134] and in ductile metal films [117,135,136]. One of the most alloyed brittle metal films on polymers found in literature is Mo. Mo has successfully been alloyed with Re, Cu, Ag, Nb, Ta, and Al [115,116,134]. For most alloy combinations, as the Mo content decreased, an improvement of the COS was observed along with a change in the crack morphology from long straight cracks to shorter zig-zag cracks. Crack morphology has already been discussed (Section 4.1); therefore, the effects on the COS and electrical resistance will be focused on here. With the different alloy compositions and the improved COS values, the change in electrical resistance with decreasing Mo content rarely follows the same trend (Figure 10). Alloying produces additional electron scattering sources in a pure metal crystal and reaches a maximum at 50% when there is total miscibility. Additionally, residual stresses will also play a role (Section 4.1). In order to have a more robust and longer lasting flexible electronic device or sensor, the electrical resistance must also be considered as a design criterion. In Figure 10, for various Mo-based alloys the resistivity as a function of the measured COS is shown for 50 nm and 500 nm thick films. For 50 nm thick films (Figure 10a), the Mo-Al alloys have an improved COS, but also have very high resistivities which make them inadequate for flexible electronic applications. On the other hand, Mo-Ta alloys have low resistivities and the highest COS values. For thicker Mo-based alloy films, the Mo-Al alloys achieve a higher COS with an adequate increased resistivity (Figure 10b). In such a graph as in Figure 10, the goal is to go as far right in the COS values while keeping the resistivity as low as possible.

The co-sputter deposited Mo-Ta alloy has been used as an interlayer with conductive Al on PI and tested under monotonic tensile straining and bending [108]. After stress tailoring the interlayer alloy, the Al/Mo-Ta bilayers performed similarly under uniaxial tensile straining with a 1% improvement in COS compared to the Al/Mo bilayers with the same thickness ratios (D:B). However, under mixed compressive tensile bending using a bending strain of 1.3%, the Al/Mo-Ta bilayers vastly outperformed the same Al/Mo bilayers. No damage or cracks were observed after 50,000 cycles in the Al/Mo-Ta bilayers, while the Al/Mo bilayers were completely damaged [108]. However, after 100 cycles using a higher bending strain of 3.1%, cracks formed in the Al/Mo-Ta bilayers [108]. This study illustrates that the material behavior can be applied to the right application, in this case bending of 1.3% or lower produces a robust, nearly unbreakable, and electrically conductive film system. Table 2 summarizes the design concepts introduced with their benefits, limitations, and possible solutions for future reference.

## 5. Summary

As presented, thin metal films can be designed to have ideal electrical and mechanical behaviors for flexible and stretchable electronics. With a clear understanding of the various thin film deposition methods and the physics behind film growth, advanced thin films with superior mechanical and functional behaviors can be created. Additionally, fabrication scalability is a concern that material design can easily address. As demonstrated for the Al/Mo-Ta system, when we know the material system’s limitations, then an application can be selected that is suitable. When the material design aspect has reached its limits, then additional device engineering concepts (i.e., meanders, wrinkling, free-standing bridges, etc.) should be exploited. It is beneficial to first utilize the materials’ design before more complex device engineering in order to use the same methods and large-scale deposition infrastructure that is currently available for the industry. The utilization of both material design and device engineering concepts will lead to synergetic affects, reduced complexity, reduced production costs, and an increase in the lifetime of a device. Changes to the available infrastructure is costly and not sustainable. The presently available understanding of thin film deposition processes, the available (in-situ) testing methods, and the gained insight into electro-mechanical degradation behavior enable the tailoring of metallic thin films for tomorrow’s flexible electronic applications. Both the robustness of the described approaches on a large-scale and their optimization require the continued combined efforts of academia and industry.

## Figures and Tables

**Table 1:** Summary of benefits, limitations, and possible solutions for electrical resistance-based and cyclic testing.

Test Type	Deposition Method	Benefit(s)	Limitation(s)	Solution(s)
Electrical resistance	Sputtering or Evaporation	Very good conductivity with commonly used parameters Measure resistance during straining and connect to mechanical damage	Difficult to determine point of failure (COS vs. DOS) Failure criteria based on increase in resistance and not damage density (or both) Lack of in-situ resistance understanding	Larger grains have lower resistance than smaller grains More research connecting electrical resistance increase with mechanical damage
	Printing	Fast deposition does not require vacuum or high temperatures No lithography necessary, direct pattern printing Measure resistance during straining and connect to mechanical damage	Generally poor electrical conductivity compared to sputtered/evaporated films Cracks can form easily at low to moderate strains	Thicker the better More/longer heat treatments to promote sintering of metal particles
Cyclic Testing	Sputtering	Test application of relevant loading conditions	No concrete failure criteria for lifetime testing No standardized bending method Integration of electrical resistance measurement into bending	Optimize film architecture Optimize ductile film microstructure to allow for grain growth Determine electrical and mechanical damage failure criteria Standardize bending methods Understand the in-situ electrical data
	Evaporation			
	Printing			

**Table 2:** Summary of benefits, limitations, and possible solutions for fabricating robust brittle or ductile metal films on polymer substrates.

Material	Deposition Method	Benefit(s)	Limitation(s)	Solution(s)
Brittle Film	Sputtering	Can improve adhesion to polymer substrate As top layer can improve fracture strength of ductile layer	Fractures at low applied strains Causes ductile films to become brittle when used as adhesion layers	Use thick ductile films with a thickness ratio of at least 1:5 (brittle:ductile) Avoid use if possible Alloy with more ductile metal Create crack arresting microstructure Increase residual stress
	Evaporation	Can improve adhesion to polymer substrate	Interface may not be thermally stable and delaminate Deposition of refractory metals may thermally overload polymer substrates	Avoid use if possible Alloy with more ductile metal
Ductile Film	Sputtering	Conductive for electrical applications Can have a thermally stable interface	When thin (≤100 nm) can easily form cracks May require brittle adhesion layers	Adapt deposition parameters to have larger grains or to alter interface structure Use very thin (≤10 nm) brittle layers to improve adhesion
	Evaporation			

## Data Availability

Any new data presented is available upon reasonable request by the authors.
